# Oscillatory brain activity associates with neuroligin-3 expression and predicts progression free survival in patients with diffuse glioma

**DOI:** 10.1007/s11060-018-2967-5

**Published:** 2018-08-09

**Authors:** Jolanda Derks, Pieter Wesseling, Ellen W. S. Carbo, Arjan Hillebrand, Edwin van Dellen, Philip C. de Witt Hamer, Martin Klein, Geert J. Schenk, Jeroen J. G. Geurts, Jaap C. Reijneveld, Linda Douw

**Affiliations:** 1 Department of Anatomy & Neurosciences, VU University Medical Center, De Boelelaan 1117, 1081 HV Amsterdam, The Netherlands; 2 VUmc CCA Brain Tumor Center Amsterdam, De Boelelaan 1117, 1081 HV Amsterdam, The Netherlands; 3 Department of Pathology, VU University Medical Center, De Boelelaan 1117, 1081 HV Amsterdam, The Netherlands; 4 Department of Pathology, Princess Máxima Center for Pediatric Oncology and University Medical Center Utrecht, Lundlaan 6, 3584 EA Utrecht, The Netherlands; 5 Department of Clinical Neurophysiology and MEG Center, VU University Medical Center, De Boelelaan 1117, 1081 HV Amsterdam, The Netherlands; 6 Department of Psychiatry, University Medical Center Utrecht, Heidelberglaan 100, 3584 CX Utrecht, The Netherlands; 7 Brain Center Rudolf Magnus, Universiteitsweg 100, 3584 CG Utrecht, The Netherlands; 8 Department of Neurosurgery, Neuroscience Campus Amsterdam, VU University Medical Center, De Boelelaan 1117, 1081 HV Amsterdam, The Netherlands; 9 Department of Medical Psychology, VU University Medical Center, De Boelelaan 1117, 1081 HV Amsterdam, The Netherlands; 10 Department of Neurology, Neuroscience Campus Amsterdam, VU University Medical Center, De Boelelaan 1117, 1081 HV Amsterdam, The Netherlands; 11 Athinoula A. Martinos Center for Biomedical Imaging/Massachusetts General Hospital, 149 13th St, Charlestown, MA 02129 USA

**Keywords:** Glioma, NLGN3, Magnetoencephalography, Neurophysiology, Tumor progression

## Abstract

**Introduction:**

Diffuse gliomas have local and global effects on neurophysiological brain functioning, which are often seen as ‘passive’ consequences of the tumor. However, seminal preclinical work has shown a prominent role for neuronal activity in glioma growth: mediated by neuroligin-3 (NLGN3), increased neuronal activity causes faster glioma growth. It is unclear whether the same holds true in patients. Here, we investigate whether lower levels of oscillatory brain activity relate to lower NLGN3 expression and predict longer progression free survival (PFS) in diffuse glioma patients.

**Methods:**

Twenty-four newly diagnosed patients with diffuse glioma underwent magnetoencephalography and subsequent tumor resection. Oscillatory brain activity was approximated by calculating broadband power (0.5–48 Hz) of the magnetoencephalography. NLGN3 expression in glioma tissue was semi-quantitatively assessed by immunohistochemistry. Peritumor and global oscillatory brain activity was then compared between different levels of NLGN3 expression with Kruskal–Wallis tests. Cox proportional hazards analyses were performed to estimate the predictive value of oscillatory brain activity for PFS.

**Results:**

Patients with low expression of NLGN3 had lower levels of global oscillatory brain activity than patients with higher NLGN3 expression (P < 0.001). Moreover, lower peritumor (hazard ratio 2.17, P = 0.008) and global oscillatory brain activity (hazard ratio 2.10, P = 0.008) predicted longer PFS.

**Conclusions:**

Lower levels of peritumor and global oscillatory brain activity are related to lower NLGN3 expression and longer PFS, corroborating preclinical research. This study highlights the important interplay between macroscopically measured brain activity and glioma progression, and may lead to new therapeutic interventions in diffuse glioma patients.

**Electronic supplementary material:**

The online version of this article (10.1007/s11060-018-2967-5) contains supplementary material, which is available to authorized users.

## Introduction

Gliomas are primary brain tumors originating from (precursors of) glial cells. Most gliomas in adult patients are characterized by diffuse infiltration of tumor cells in the surrounding brain parenchyma. These so-called diffuse gliomas have widespread effects on neurophysiological functioning as measured with magnetoencephalography (MEG) and electroencephalography (EEG): both increased low frequency activity [1, 2] and altered synchronization of activity [3–5], which is commonly referred to as functional connectivity, have been reported. Moreover, particularly altered functional connectivity has deleterious correlates: glioma patients with higher delta (0.5–4 Hz) and/or theta (4–8 Hz) band connectivity show poorer cognitive performance [6–8], and more often suffer from epileptic seizures [9, 10].

Emerging evidence shows that the relationship between altered neurophysiological functioning and glioma is more reciprocal than previously thought. A series of in vivo and in vitro animal experiments show that increased neuronal spiking of neurons surrounding the tumor significantly enhances tumor growth. Neuroligin-3 (NLGN3), a cell adhesion protein on the postsynaptic membrane that is secreted through neuronal activity, is identified as a key contributor to this process [11]. More recently, it is shown that blocking the release of NLGN3 results in diminished tumor growth in animal models of high-grade glioma [12].

The relation between neuronal activity, NLGN3 expression and tumor progression has not been established in a clinical population of patients with diffuse glioma, which hampers clinical exploitation of these obtained fundamental insights. Therefore, we investigated oscillatory brain activity, operationalized as broadband MEG oscillatory power [13]. MEG measures the activity of large populations of neurons oscillating in synchrony and is a non-invasive technique to measure neuronal activity [14]. Moreover, NLGN3 expression was assessed through immunohistochemistry of resected glioma tissue. We hypothesized that patients with lower peritumor and global levels of oscillatory brain activity have lower NLGN3 expression and a longer progression free survival (PFS) than patients with higher levels of oscillatory brain activity.

## Methods

### Patients

Patients visiting the VUmc CCA Brain Tumor Center Amsterdam with suspected de novo diffuse glioma between 2010 and 2012 could participate. Part of this cohort has been described previously [8, 15, 16]. Inclusion criteria were (1) age over 17 years old, (2) histopathologically confirmed World Health Organization (WHO) 2007 grade II, III or IV diffuse glioma [17], (3) at least one seizure (ensuring homogeneity of the cohort in terms of tumor-related epilepsy), and (4) ability to complete neuropsychological testing (data not used in this study). All patients underwent (sub)total tumor resection (as investigated by manual segmentation of remnant tumor tissue using anatomical imaging [LD]), with all patients having at least 76% of the glioma volume resected. Because of low variation in this cohort, extent of resection was not further explored as a covariate. Patients with prior neurological and psychiatric diseases or previous craniotomies were not eligible for this study.

Tumor progression was based on agreement within the multidisciplinary tumor board of the VUmc CCA Brain Tumor Center Amsterdam taking radiological and clinical information into account. Time to progression was counted from the date of the preoperative MEG in weeks. Patients without progression within the follow-up time frame (June 2017) were censored as of their last contact date.

In addition, 24 sex- and age-matched healthy controls were included to normalize oscillatory brain activity. This study was approved by the ethical review board of the VU University Medical Center, informed consent was obtained from all individual participants included in the study.

### Magnetoencephalography

Oscillatory brain activity was measured non-invasively by MEG [14]. The average time between MEG recording and surgery was 8.4 weeks. Recording and preprocessing methods have been published before [15, 16, 18] and are explained in detail in the supplementary material. In short, patients underwent a 5-min eyes-closed resting state recording using a 306-channel MEG system (Elekta Neuromag Oy, Helsinki, Finland). Patients’ anatomical MRIs were co-registered to the MEG and 78 cortical parcels were selected for analyses [19, 20]. The MEG time-series were then reconstructed using a scalar beamformer implementation (Elekta Neuromag Oy, version 2.1.28). As a non-invasive measure of neuronal activity, we calculated broadband (0.5–48 Hz) oscillatory power [13].

### Peritumor, global, and non-tumor oscillatory activity

**Fig. 1:**
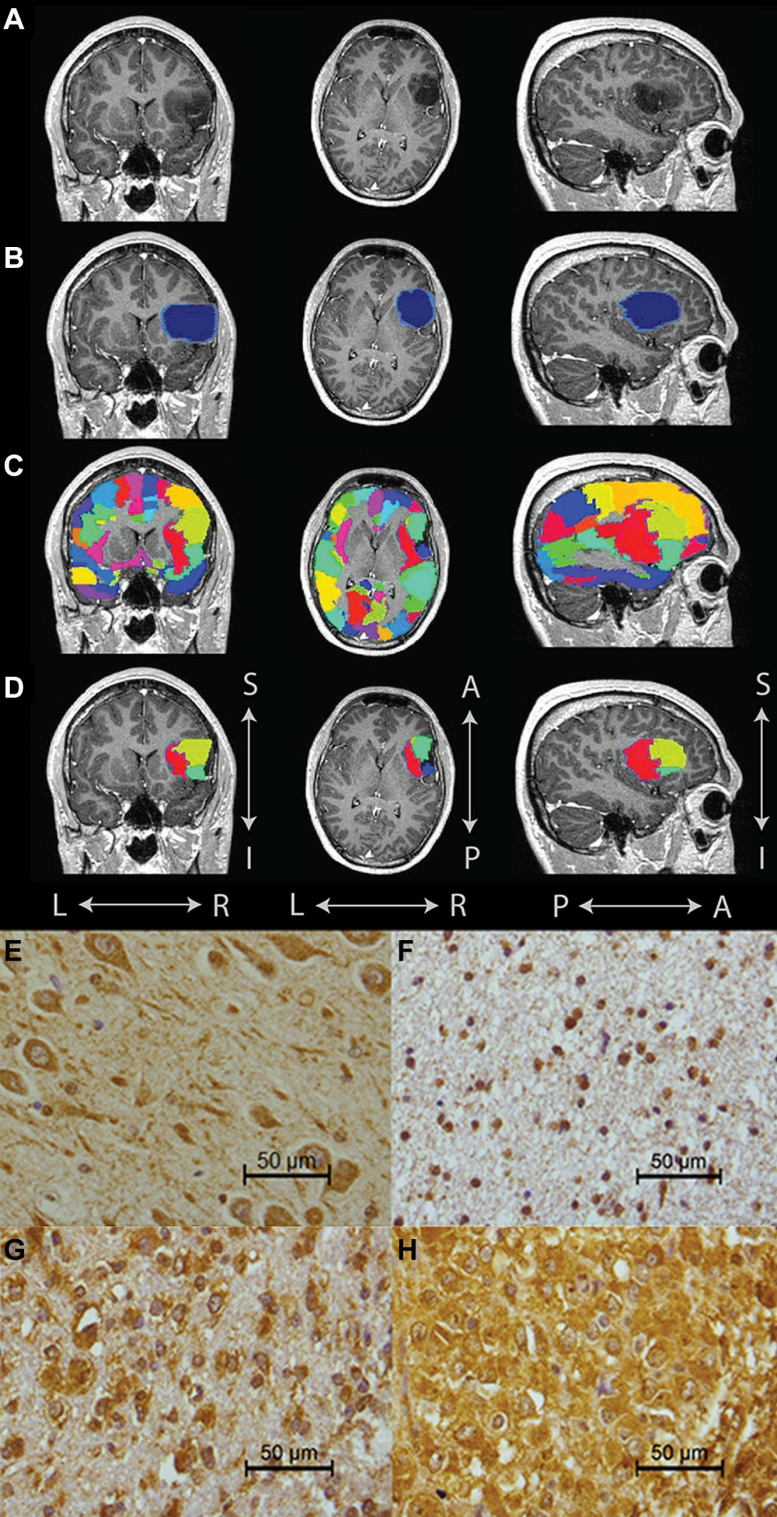
Tumor mask, cortical areas and NLGN3 immunohistochemistry. Example of tumor mask, peritumor and global oscillatory activity regions (**a**–**d**): **a** indicates an exemplar T1 weighted scan (post gadolinium) with low-grade glioma in the left frontal region, **b** depicts the manually segmented tumor mask (dark blue) and peritumor area (light blue), **c** displays the 78 cortical areas used to calculate global oscillatory brain activity in color, and **d** contains the atlas regions that fall within the tumor and surrounding tumor area used to calculate peritumor oscillatory brain activity. Examples of tissue expressing neuroligin-3 (**e**–**h**): **e** hippocampus with moderate to strong staining of neuronal cell bodies and their processes, **f** low NLGN3 expression outside the cell nuclei, **g** moderate NLGN3 expression especially in tumor cell cytoplasm, and **h** high NLGN3 expression with strong staining of tumor cells as well as extensive staining of neuropil in between. *A* anterior, *I* inferior, *L* left, *NLGN3* neuroligin-3, *P* posterior, *R* right, *S* superior

MR images were used to manually draw the tumor (L.D.; Fig. 1a) to create a MRI tumor mask [21]. Next, the tumor mask was dilated two times with the default FSL kernel (Fig. 1b). The 78 atlas regions were projected onto patients’ individual scans (Fig. 1c). The average broadband power of regions overlapping with the dilated tumor mask formed a ‘peritumor’ oscillatory activity measure per patient (Fig. 1d). Global oscillatory activity was calculated by averaging the absolute power values of all 78 regions per patient. Non-tumor oscillatory activity was determined by averaging the absolute power values of all regions except those within the dilated tumor mask. In order to compare these values across patients, individual values for peritumor, global and non-tumor oscillatory activity were converted to z-scores using the absolute power values of the entire patient cohort. For global oscillatory power only, z-scores were also calculated based on the values of the healthy controls, in order to allow for comparison of the hazard ratio associated with higher levels of oscillatory activity as compared to healthy levels.

### Tissue micro array and immunohistochemistry

Glioma tissue acquired during surgery (inspected by J.D. and P.W.), was processed in a tissue micro array (TMA) with 0.6 mm diameter cores and sections were stained for NLGN3 by immunohistochemistry (see supplementary material; Fig. 1e–h). In line with information of the protein atlas (https://www.proteinatlas.org/ENSG00000196338-NLGN3/tissue), examination of the NLGN3-stained slides revealed abundant expression in neurons. A section of hippocampal tissue was therefore used as positive control (Fig. 1e). Three investigators (J.D., J.J.G.G., G.J.S.) semi-quantitatively assessed the NLGN3 staining. For each patient, the core with representative, viable tumor tissue with the highest NLGN3 expression was used for classification of the patients in the low, moderate or high NLGN3 expression group. To classify the cores into low, moderate or high NLGN3 expression, both the intensity and surface area of DAB positivity in the cytoplasm of all cells, as well as DAB in the neuropil were considered (Fig. 1f–h). Some aspects of the samples were not taken into account since they might not accurately represent glioma tissue or NLGN3 expression. These exclusions concerned (1) tissue containing large necrotic areas, (2) tissue without proper quality due to mechanical or technical difficulties, (3) tissue with ample preexistent grey matter (as neuronal cell bodies contain high amounts of NLGN3). Furthermore, staining of the nuclei was ignored because variable and sometimes strong nuclear staining was present in many tumor cores as well as in non-neoplastic tissue samples and may represent non-specific staining because NLGN3 protein can be expected to be located outside the cell nuclei.

In order to retrospectively assess the isocitrate dehydrogenase (*IDH*) mutational status of the samples used for this study the TMAs were also immunohistochemically stained for the *IDH1* R132H (dilution 1:1250, mouse monoclonal, clone H09; dianova GmbH, Hamburg, Germany) mutant protein (a simple, routinely used immunohistochemical tool for detection of about 90% of all *IDH*-mutant gliomas).

### Possible confounders of oscillatory brain activity and progression free survival

Several factors may influence oscillatory activity and/or PFS and were included as confounders. For PFS these possible confounders were WHO 2007 tumor grade (grade II or grade III–IV) [17, 22, 23], Karnofsky performance status (KPS) (≤ 80 or ≥ 90) [24], and age (over or under 40 years) [23]. Regarding molecular information, *IDH1* mutation status is an important prognostic factor and was investigated as a confounder [25]. Moreover, in the case of an *IDH*-mutant glioma, the absence or presence of 1p/19q codeletion holds additional prognostic information [25]. In a subset of this cohort, 1p/19q codeletion was determined using loss-of-heterozygosity analysis for clinical purposes. Of note, according to the revised fourth edition of the WHO classification of CNS tumors (published in 2016), diffuse gliomas can be classified in three groups: (1) *IDH*-mutant with 1p/19q codeletion (‘canonical’ oligodendrogliomas; most favorable prognosis), (2) *IDH*-mutant but 1p/19q-non-codeleted (intermediate prognosis), and (3) *IDH*-wildtype (poorest prognosis, often glioblastomas (GBM)) [26, 27]. *IDH1*-mutant astrocytoma patients without assessment of 1p/19q status were retrospectively labeled as 1p/19q-non-codeleted. Other confounders that were explored were adjuvant treatment (radiotherapy plus chemotherapy, radiotherapy alone, or no adjuvant treatment), tumor histology (oligodendroglioma, oligoastrocytoma, astrocytoma), midline crossing of the tumor (i.e. bilateral localization) and tumor diameter (diameter ≤ 6 cm or > 6 cm in any direction) [23, 24]. We also considered localization in eloquent areas, i.e. areas that if resected would result in loss of function, as a confounder of PFS [24]. Furthermore, oscillatory brain activity may be influenced by age (continuous) [28, 29], tumor volume [1] and patients’ head size (based on MRI using SIENA from the FSL toolbox), as larger heads are usually closer to the sensors [14]. Finally, sex was also investigated as a confounder.

### Statistical analyses

Statistical analyses were performed using the PASW Statistics package (version 22.0.0.0, IBM Corp., Armonk, NY, USA) and Matlab (version R2012.a, Mathworks, Natick, MA, USA). Peritumor, global, and non-tumor oscillatory brain activity were normally distributed, tested by Kolmogorov–Smirnov tests (P < 0.05). A Student’s *t* test was used to test differences in global oscillatory brain activity between patients and healthy controls. To test differences in peritumor, global and non-tumor broadband oscillatory brain activity levels between the NLGN3 expression groups, three Kruskal–Wallis tests were performed, each followed by three post hoc Mann–Whitney U pairwise comparisons.

Cox proportional hazards models were used to test whether peritumor, global (z-scores based on the distribution of the patients as well as on the distribution of the control cohort), non-tumor broadband oscillatory brain activity and NLGN3 expression were significant predictors of PFS.

Leave-one-out and permutation analyses were performed to validate the results regarding global broadband oscillatory activity and PFS. For the leave-one-out analyses, 24 Cox proportional hazard models were computed, each time excluding one patient from the entire cohort of 24 patients. For the permutation analyses, global oscillatory brain activity values were randomly shuffled between patients 1000 times, after which P-values for the Cox proportional hazard model were obtained for each permutation separately. From these analyses, a cohort-specific distribution of P-values was created. The experimental P-value was tested against this distribution with a 0.05 alpha cut-off.

Additionally, thirteen multivariate models were created to test the effect of possible confounders. Each model included global broadband oscillatory brain activity with one covariate, due to the small sample size.

A significance level of 0.05 (two-tailed) was used.

## Results

### Patient characteristics

Twenty-four glioma patients with a mean age of 39 ± 10.53 (SD) years were included (Table 1). Sixteen patients had a WHO grade II and six patients a WHO grade III diffuse glioma, while in two patients the diagnosis was GBM (astrocytoma WHO grade IV). In 21 patients, *IDH1* mutation status was known and for nine patients 1p/19q codeletion information was available. Five patients had an *IDH1*-mutant astrocytoma and were, based on that information, classified as having no 1p/19q codeletion. This yielded four patients with an *IDH1*-mutant, 1p/19q-codeleted tumor, seven patients with an *IDH1*-mutant, 1p/19q-non-codeleted tumor, and six patients with most likely an *IDH*-wildtype tumor (see Table 1). Seventeen patients showed progression within the follow-up timeframe, with a median PFS of 87 ± 86 weeks. Global oscillatory brain activity did not differ between healthy controls and patients (t(46) = − 0.74, P = 0.465).

**Table 1:** Patient characteristics

M/F	Age	WHO 2007 diagnosis	*IDH1* status	Tumor location	AED use	Tumor volume (cm^3^)	EOR (%)	Treatment after resection until progression	KPS	PFS or last follow-up (weeks)	OBA (z-score)	NLGN3 expression
M	48	OII	Mut	R-FrT	VPA	14.93	100	–	70	301	− 1.85	Low
F	26	AIII	Mut^b^	L-T	VPA	147.33	99	Re-resection^a^, RTH, TMZ	90	**224**	− 1.49	Low
F	26	AIII	WT^b^	R-Fr	VPA	35.02	93	RTH	100	321	− 1.46	Low
M	41	OAII	Mut	L-P	VPA	35.95	94	RTH	90	292	− 0.84	Low
M	37	OIII	Mut^c^	R-Fr	VPA	44.74	100	RTH, PCV	100	219	− 0.69	High
F	53	AII	NA	L-T	LEV	94.65	98	RTH	100	**265**	− 0.62	NA
M	47	AIII	WT^b^	R-P	–	41.30	76	RTH, TMZ	100	**54**	− 0.47	Low
F	28	OII	Mut^b^	LT	LEV	61.51	97	–	100	**66**	− 0.37	Low
M	28	AII	WT	L-Fr	LEV	68.07	96	–	100	**45**	− 0.26	Low
M	43	AII	Mut	L-T	VPA	53.41	100	–	80	305	− 0.24	Low
F	37	AII	Mut	L-T	CBZ	107.14	100	–	100	**65**	− 0.23	Low
M	36	OIII	WT^c^	L-Fr	LEV	27.61	97	RTH	100	293	− 0.15	High
M	18	AII	Mut	L-Fr	VPA	36.46	92	–	100	**251**	− 0.14	Low
M	35	OAII	Mut^c^	R-P	LEV	52.85	100	–	100	**29**	− 0.12	Moderate
M	30	AII	WT	R-T	PHT, LEV	85.03	91	–	100	**156**	0.23	Low
M	46	GBM	WT	L-Fr	VPA	123.05	99	RTH, TMZ	90	**39**	0.31	High
M	27	AII	Mut	L-P	VPA	48.54	92	–	90	**93**	0.33	Low
M	37	GBM	NA	L-Fr	LEV	56.44	100	RTH, TMZ	100	**87**	0.46	NA
F	48	AII	Mut	R-T	VPA	72.42	100	–	100	**106**	0.50	Moderate
M	52	OII	Mut^c^	L-T	–	53.22	97	–	100	**250**	0.57	Moderate
M	58	OIII	Mut^c^	L-Fr	LEV	35.20	100	RTH, PCV	90	200	0.74	Moderate
M	30	AII	NA	L-Fr	PHT	35.27	100	–	90	**54**	1.69	NA
M	46	OAII	Mut	R-T	VPA	76.58	88	–	100	**164**	1.72	Moderate
M	50	AII	WT	L-T	–	47.68	95	–	80	**8**	2.38	Moderate

PFS is displayed in bold. Z-scores of global oscillatory brain activity are based on mean and standard deviation of the patient cohort

*A* astrocytoma, *AED* anti-epileptic drug, *CBZ* carbamazepine, *EOR* extend of resection, *F* female, *Fr* frontal, *GBM* glioblastoma, *KPS* Karnofsky performance statuts, *L* left, *LEV* levetiracetam, *M* male, *Mut* mutation, *NA* not available, *NLGN3* neuroligin-3, *O* oligodendroglioma, *OA* oligoastrocytoma, *OBA* oscillatory brain activity, *P* parietal, *PCV* procarbazine lomustine vincristine, *PHT* phenytoin, *R* right, *RTH* radiotherapy, *T* temporal, *TMZ* temozolomide, *VPA* valproate, *WHO* World Health Organization, *WT* wildtype

^a^Re-resection was not based on tumor progression

^b^Patients without 1p/19q codeletion

^c^Patients with 1p/19q codeletion

### Expression of NLGN3 is associated with oscillatory brain activity

Expression of NLGN3 could be determined in 21 patients, since tissue of three patients was unavailable. Twelve patients were classified as having low expression of NLGN3, six patients had moderate expression and three patients had high expression.

**Fig. 2:**
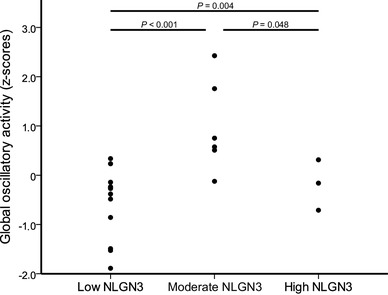
Z-scores of global oscillatory activity in the three NLGN3 expression groups. Patients with lowest NLGN3 expression showed the lowest global oscillatory activity. *NLGN3* neuroligin-3

Peritumor (H(2) = 9.07, P = 0.011), non-tumor (H(2) = 9.09, P = 0.011) and global (H(2) = 11.13, P = 0.004) oscillatory brain activity levels were significantly different according to NLGN3 expression (Fig. 2). Together, these results indicate that not only activity of the tumor region, but also global activity (with and without the tumor included) associates with NLGN3 expression. Post-hoc analyses showed that patients with moderate NLGN3 expression had significantly higher levels of peritumor (U = 7, P = 0.005), global (U = 2, P < 0.001) and non-tumor (U = 5, P = 0.002) oscillatory brain activity than patients with low NLGN3 expression. Levels of peritumor (U = 0, P = 0.025) and global (U = 1, P = 0.048) oscillatory brain activity between patients with high NLGN3 expression and moderate NLGN3 expression were also significantly different: oscillatory activity was lower in the three patients with the highest expression levels.

NLGN3 expression (low and moderate only) was not a predictor of PFS (Hazard Ratio (HR) 0.54, 95% confidence interval (CI) 0.17–1.7, P = 0.300).

### Oscillatory brain activity predicts progression free survival

**Fig. 3:**
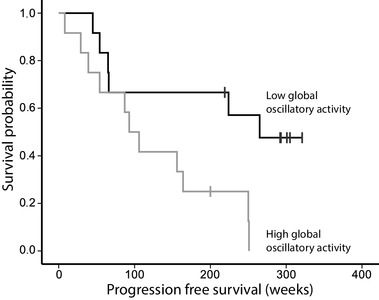
Kaplan Meier survival curves. Patients with low global oscillatory brain activity (N = 12, black line) had longer progression free survival than patients with high global oscillatory brain activity (N = 12, grey line), based on a median split of the entire cohort

Univariate Cox proportional hazard models revealed a significant hazard ratio of peritumor (HR 2.17, CI 1.23–3.85, P = 0.008), global (HR 2.10, CI 1.22–3.63, P = 0.008) and non-tumor (HR 2.05, CI 1.18–3.58, P = 0.011) oscillatory brain activity for PFS, indicating that lower levels of oscillatory brain activity were associated with longer PFS. The result regarding global oscillatory brain activity and PFS was replicated by the leave-one-out analyses, with P-values ranging between 0.004 and 0.036, and remained significant when creating a sample-specific P-value distribution through permutation analyses. Furthermore, comparable results were found using z-scores based on the mean and SD of healthy controls (HR 1.93, CI 1.19–3.13, P = 0.008). To visualize this difference in PFS for different levels of global oscillatory brain activity, we applied a median split on the global oscillatory activity values and created Kaplan Meier curves for low and high levels of oscillatory brain activity (Fig. 3, Supplementary Table S1).

The high oscillatory brain activity group included the two GBM patients, which may have skewed these analyses. We therefore repeated the Cox proportional hazard analysis for global oscillatory activity and PFS without the two GBM patients, which yielded comparable results (HR 2.12, CI 1.20–3.79, P = 0.010).

### Confounding factors do not influence the predictive value of global oscillatory activity for PFS

Global oscillatory activity remained a significant predictor of PFS in all analyses when taking several confounders into account (Table 2). Three confounders (almost) reached significance in addition to global oscillatory activity: molecular status was a trend level predictor of PFS (HR 2.33, CI 0.99–5.51, P = 0.054), tumor histology (HR 2.23, CI 1.03–4.82, P = 0.041) and tumor volume were significant predictors of PFS (HR 1.02, CI 1.00–1.04, P = 0.019). Of note, only six patients had a tumor located in an eloquent area, and radiologically none of the patients had a tumor crossing the midline.

**Table 2:** Influence of possible confounders

Confounders	Global oscillatory activity			Confounder		
	HR	CI	P-value	HR	CI	P-value
Age (≤ 40)	2.40	1.33–4.36	0.004**	2.20	0.78–3.22	0.138
Age (years continuous)	2.33	1.31–4.13	0.004**	0.97	0.93–1.02	0.210
Gender	2.49	1.35–4.61	0.004**	0.44	0.13–1.47	0.183
KPS (≤ 80)	2.04	1.17–3.58	0.013*	0.49	0.06–3.88	0.497
WHO grade	2.05	1.17–3.59	0.013*	1.23	0.39–3.93	0.723
Histology	2.37	1.32–4.25	0.004**	2.23	1.03–4.82	0.041*
*IDH1* mutation status (N = 18)	2.13	1.14–3.97	0.018*	2.10	0.66–6.69	0.209
*IDH1* mutation status combined with 1p/19q codeletion (N = 18)	2.88	1.14–7.28	0.025*	2.33	0.99–5.51	0.054
Adjuvant treatment	2.09	1.17–3.74	0.013*	1.02	0.52–2.00	0.958
Tumor volume (cm^3^)	2.29	1.33–3.93	0.003**	1.02	1.00–1.04	0.019*
Tumor diameter (< 6 cm)	2.02	1.16–3.51	0.013*	0.68	0.26–1.82	0.445
Non-eloquent area	2.28	1.28–4.06	0.005**	0.52	0.17–1.60	0.256
Head size	2.23	1.24–4.01	0.007**	1.16	0.69–1.93	0.580

Results of Cox proportional hazard models for the thirteen separate multivariate analyses using global oscillatory activity combined with each of the confounders

*CI* 95% confidence interval, *HR* hazard ratio, *KPS* Karnofsky performance status, *WHO* World Health Organization

*P < 0.05; ** *P* < 0.01

## Discussion

Lower peritumor oscillatory brain activity was associated with lower NLGN3 expression and was predictive of longer PFS in a cohort of newly-diagnosed diffuse glioma patients. The predictive value of oscillatory brain activity was valid in the peritumor region, corroborating previous work performed in an animal model of glioma [11]. Moreover, the same results were obtained for activity across the entire brain, extending the current knowledge based on this interesting association between activity, NLGN3 and ultimately survival.

Neuroligin-3 is a tumor growth promoting protein and is secreted through neuronal activity and induces NLGN3 expression in glioma cells [11, 12]. NLGN3 is not only secreted through neuronal spiking, but also by activity of oligodendrocyte precursor cells [12, 30]. However, only NLGN3 secreted through neuronal spiking has been shown to promote glioma growth [12]. This emphasizes the specificity of brain activity in relation to NLGN3 expression, as corroborated by the association between oscillatory brain activity and NLGN3 expression in our study. As hypothesized, patients with lower expression levels of NLGN3 showed lower levels of oscillatory brain activity than patients with higher expression levels of NLGN3.

Contrary to our expectations, patients with the highest NLGN3 expression levels did not show the highest oscillatory brain activity levels and could be due to sampling issues. Conversely, this could be the result of some sort of inverted U-shape in the association between global oscillatory activity and NLGN3 expression, or of a particular subtype of glioma in this group. Interestingly, two patients with the highest NLGN3 expression had a histological diagnosis of WHO grade III oligodendroglioma, and one can speculate that in these tumors the NLGN3 expression level is relatively high irrespective of the level of NLGN3 that is released by neuronal activity [31]. However, the fact that this group with the highest expression only contains 3 patients precludes drawing firm conclusions for this particular category.

Our results suggest that broadband oscillatory activity may indeed be used as an operationalization of neuronal activity in the context of glioma, for instance to monitor tumor growth. One should keep in mind though that MEG does not measure action potentials directly, but assesses post-synaptic potentials that are indirectly related to neuronal spiking. This means that we cannot be sure that our findings are specific to neuronal spiking. Nevertheless, MEG oscillatory activity remains the most direct non-invasive measure of neuronal activity available at this time [14], and should be further explored as a tool for prognostic purposes.

In addition, our results suggest that (global) brain activity may be a viable treatment target in glioma, possibly in addition to inhibition of NLGN3 secretion [12]. For instance, anti-epileptic drugs diminish neuronal excitability and might therefore slow down tumor growth: several studies have shown survival benefits in GBM patients on anti-epileptic drugs [32–35], although a recent meta-analysis did not confirm this association [36]. Of course, the beneficial effect might be dose dependent [37]. Additionally, the effect may relate to individual differences in the previously unknown and unmeasured levels of neuronal activity, for which we here describe a possible biomarker that may be validated as such in future work.

Of note, the patient cohort in this study does not fully represent the diffuse glioma population in general. This is probably due to the participation bias, patients had to be in a relative good condition in order to be able to undergo MEG and neuropsychological testing. Patients included in our study had a generally high KPS, more frequently a low grade diffuse gliomas, and were relatively young. Another point of discussion is that NLGN3 may well be expressed in a heterogeneous manner in the glioma tissue [38]. Considering the glioma tissue on the TMA, sampling a small area of the tumor might result in inaccurate estimates of expression levels.

In conclusion, lower levels of oscillatory brain activity are associated with lower NLGN3 expression and predict longer PFS. Our study highlights the relevance of neuronal activity for tumor progression in a clinical glioma population, and could be a next step towards improved prognosis and additional treatment strategies.

## Electronic supplementary material

Below is the link to the electronic supplementary material.

Supplementary material 1 (DOCX 26 KB)
