# Supplementary material for: Oscillatory brain activity associates with neuroligin-3 expression and predicts progression free survival in patients with diffuse glioma
Source: J Neurooncol. 2018 Aug 9;140(2):403–12. doi: 10.1007/s11060-018-2967-5 (PMC6244774; doi:10.1007/s11060-018-2967-5)
Supplement: Supplementary file 1 — Supplementary material 1 (DOCX 26 KB) [file 11060_2018_2967_MOESM1_ESM.docx]

**Supplementary Materials**

**Oscillatory brain activity associates with neuroligin-3 expression and predicts progression free survival in patients with diffuse glioma**

Jolanda Derks^1,2^, Pieter Wesseling^2,3,4^, Ellen W. S. Carbo^1^, Arjan Hillebrand^5^, Edwin van Dellen^6,7^, Philip C. de Witt Hamer^8^, Martin Klein^9^, Geert J. Schenk^1^, Jeroen J. G. Geurts^1^, Jaap C. Reijneveld^10^, Linda Douw^1,2,11^

^1^Department of Anatomy & Neurosciences, VU University Medical Center, Amsterdam, The Netherlands. ^2^VUmc CCA Brain Tumor Center Amsterdam, Amsterdam, The Netherlands. ^3^Department of Pathology, VU University Medical Center, Amsterdam, The Netherlands. ^4^Department of Pathology, Princess Máxima Center for Pediatric Oncology and University Medical Center Utrecht, Utrecht, The Netherlands. ^5^Department of Clinical Neurophysiology and MEG Center, VU University Medical Center, De Boelelaan 1117, 1081 HV Amsterdam, The Netherlands. ^6^Department of Psychiatry, University Medical Center Utrecht, Heidelberglaan 100, 3584 CX, Utrecht, The Netherlands. ^7^Brain Center Rudolf Magnus, Universiteitsweg 100, 3584 CG, Utrecht, The Netherlands. ^8^Department of Neurosurgery, Neuroscience Campus Amsterdam, VU University Medical Center, De Boelelaan 1117, 1081 HV Amsterdam, The Netherlands. ^9^Department of Medical Psychology, VU University Medical Center, De Boelelaan 1117, 1081 HV Amsterdam, The Netherlands. ^10^Department of Neurology, Neuroscience Campus Amsterdam, VU University Medical Center, De Boelelaan 1117, 1081 HV Amsterdam, The Netherlands. ^11^Athinoula A. Martinos Center for Biomedical Imaging / Massachusetts General Hospital, 149 13th St, Charlestown, MA 02129, US.

**Corresponding author**

Linda Douw, Department of Anatomy & Neurosciences, VU University Medical Center, De Boelelaan 1117, 1081 HV Amsterdam, The Netherlands. Tel: +31 621137147, email: L.Douw@vumc.nl

Journal of Neuro-Oncology

**Methods**

**Magnetoencephalography**

Oscillatory brain activity was measured non-invasively by MEG [1]. The average time between MEG recording and surgery was 8.4 weeks. Recording and preprocessing methods have been published in detail before [2–4]. In short, patients underwent a 5-minute eyes-closed resting state recording using a 306-channel MEG system (Elekta Neuromag Oy, Helsinki, Finland). Data were sampled at 1250 Hz and an anti-aliasing filter (410 Hz) and high pass filter (0.1 Hz) were applied online. The outline of the scalp and 4 head localization coils were digitized using a 3D digitizer (3Space Fastrak, Polhemus, Colchester, VT, USA). Malfunctioning channels were excluded based on visual inspection (E.C., E.V.D.). Offline artefact removal was performed using the temporal extension of Signal Space Separation (tSSS) in MaxFilter software (Elekta Neuromag Oy, version 2.2.15) [5].

Patients’ anatomical MRIs were co-registered to the MEG by matching the scalp surface with a co-registration accuracy of approximately 4 mm and then spatially normalized to a template MRI [6]. Using the automated anatomical labeling (AAL) atlas [7], 78 cortical parcels were selected for analyses after inverse transformation to patient’s normalized MRI [8, 9]. The MEG time-series were then reconstructed using a scalar beamformer implementation (Elekta Neuromag Oy, version 2.1.28). For each parcel, we selected the time-series of the lower alpha (8-10 Hz) peak voxel to represent a parcel by a single time-series. Lower alpha band oscillations are predominantly present during eyes-closed resting-state, yielding signals with the highest signal-to-noise ratio. This approach yielded 78 time-series that were visually inspected (E.C., J.D.) per epoch of 13.11 seconds. The first five artefact-free epochs per patient were selected for further analyses.

As a non-invasive measure of neuronal activity, we calculated broadband (0.5-48 Hz) oscillatory power [10]. Time-series were first downsampled four times followed by a fast Fourier transform to decompose the time series into the broadband frequency using Brainwave v0.9.133.1 (http://home.kpn.nl/stam7883/brainwave.html). Absolute broadband power was then averaged over the five selected epochs for each patient.

**Peritumor, global, and non-tumor oscillatory activity**

First, T1-weighted and FLAIR/T2-weighted MR images obtained before and after injection of gadolinium were used to manually draw the tumor (L.D.; Fig. 1a) on the T1-weighted images to create a MRI tumor mask [11]. Next, the tumor mask was dilated two times with the default FSL kernel (3x3x3 box; Fig. 1b). The 78 atlas regions were projected onto patients’ individual scans (Fig. 1c) and regions overlapping with the dilated tumor mask were determined. The average broadband power of these regions formed a ‘peritumor’ oscillatory activity measure per patient (Fig. 1d). Global oscillatory activity was calculated by averaging the absolute power values of all 78 regions per patient. Non-tumor oscillatory activity was determined by averaging the absolute power values of all regions except those within the dilated tumor mask. In order to compare these values across patients, individual values for peritumor, global and non-tumor oscillatory activity were converted to z-scores using the mean and standard deviation of the entire patient cohort. For global oscillatory power only, z-scores were also calculated based on the mean and standard deviation of the healthy controls, in order to allow for comparison of the hazard ratio associated with higher levels of oscillatory activity as compared to healthy levels of oscillatory brain activity.

**Tissue micro array and immunohistochemistry**

Paraffin embedded glioma tissue blocks acquired during surgery were used for NLGN3 immunohistochemistry. The tissue parts containing tumor (inspected by J.D. and P.W.) were processed in a tissue micro array (TMA) with 0.6 mm diameter cores. Cores were taken in threefold, except for one subject, for which two cores were taken due to limited availability of glioma tissue.

TMA sections of 5 µm were deparaffinized in xylene and ethanol. Antigen retrieval was done by incubating the sections in 10mM citrate buffer (pH 6.0) for thirty minutes in a steamcooker and cooled until room temperature. Sections were rinsed in tris-buffered saline (TBS) (pH 7.6) before endogenous peroxidase was blocked by a solution of TBS, 0.3% hydrogen peroxidase and 0.1% sodium azide for 30 minutes and afterwards rinsed in TBS. To block non-specific binding sites, sections were incubated in 5% non-fat dry milk in TBS with 0.5% Triton X-100 (TBS-T). The primary antibody (mouse monoclonal, ab186307, Abcam, Cambridge, UK) against NLGN3 was diluted (1:500) in TBS-T containing 1% non-fat dry milk followed by incubation of the sections for one hour at room temperature. Primary antibody was rinsed from the sections with TBS before incubation (30 minutes) in EnVision mouse HRP (undiluted, DAKO, Glostrup, Denmark). The sections were rinsed with TBS and Tris-buffer (pH 7.6) before the antibody complex was visualized with 3,3’-diaminobenzidine (DAB) (DAKO, Glostrup, Denmark). Next, sections were rinsed with Tris-buffer and water and sections were stained with haematoxyline to visualize nuclei. The sections were rinsed in running tap water before they were dehydrated with ethanol, cleared in xylene and coverslipped with Entellan (Merck, Darmstadt, Germany).

**References**

1. Hamalainen M, Hari R, Ilmoniemi RJ, et al (1993) Magetoenchepalography-Theory, Instrumentation, and Applications to Noninvasive Studies of the Working Human Brain. Rev Mod Phys 65(2):413–497. doi: 10.1103/RevModPhys.65.413

2. van Dellen E, Douw L, Hillebrand A, et al (2014) Epilepsy surgery outcome and functional network alterations in longitudinal MEG: a minimum spanning tree analysis. Neuroimage 86:354–63. doi: 10.1016/j.neuroimage.2013.10.010

3. Carbo EWS, Hillebrand A, van Dellen E, et al (2017) Dynamic hub load predicts cognitive decline after resective neurosurgery. Sci Rep 7:42117. doi: 10.1038/srep42117

4. van Dellen E, de Witt Hamer PC, Douw L, et al (2013) Connectivity in MEG resting-state networks increases after resective surgery for low-grade glioma and correlates with improved cognitive performance. NeuroImage Clin 2:1–7. doi: 10.1016/j.nicl.2012.10.007

5. Taulu S, Hari R (2009) Removal of magnetoencephalographic artifacts with temporal signal-space separation: demonstration with single-trial auditory-evoked responses. Hum Brain Mapp 30:1524–34. doi: 10.1002/hbm.20627

6. Whalen C, Maclin EL, Fabiani M, Gratton G (2008) Validation of a method for coregistering scalp recording locations with 3D structural MR images. Hum Brain Mapp 29:1288–301. doi: 10.1002/hbm.20465

7. Tzourio-Mazoyer N, Landeau B, Papathanassiou D, et al (2002) Automated anatomical labeling of activations in SPM using a macroscopic anatomical parcellation of the MNI MRI single-subject brain. Neuroimage 15:273–89. doi: 10.1006/nimg.2001.0978

8. Hillebrand A, Barnes GR, Bosboom JL, et al (2012) Frequency-dependent functional connectivity within resting-state networks: an atlas-based MEG beamformer solution. Neuroimage 59:3909–21. doi: 10.1016/j.neuroimage.2011.11.005

9. Gong G, He Y, Concha L, et al (2009) Mapping anatomical connectivity patterns of human cerebral cortex using in vivo diffusion tensor imaging tractography. Cereb Cortex 19:524–536. doi: 10.1093/cercor/bhn102

10. Manning JR, Jacobs J, Fried I, Kahana MJ (2009) Broadband Shifts in Local Field Potential Power Spectra Are Correlated with Single-Neuron Spiking in Humans. J Neurosci 29:13613–13620. doi: 10.1523/JNEUROSCI.2041-09.2009

11. Derks J, Dirkson AR, de Witt Hamer PC, et al (2017) Connectomic profile and clinical phenotype in newly diagnosed glioma patients. NeuroImage Clin 14:87–96. doi: 10.1016/j.nicl.2017.01.007

**Supplementary Table S1** Demographic information according to the Kaplan Meier curves of Fig. 3

| Confounders | Low global oscillatory activity (N=12) | High global oscillatory activity (N=12) |
| --- | --- | --- |
| Progression during follow-up (Yes/No) | 6/6 | 11/1 |
| Tumor type (A/O/OA) | 7/4/1 | 6/2/2 |
| WHO grade (II/III/IV) | 7/5/0 | 9/1/2 |
| *IDH1* mutation (wildtype/mutation/NA) | 4/7/1 | 3/7/2 |
| Tumor volume cm^3^ (mean) | 60.97 | 60.23 |
| KPS (70-80/90-100) | 2/10 | 1/11 |

Eleven out of twelve patients in the high oscillatory activity group showed progression within follow-up, compared to six out of twelve patients in the low oscillatory activity group. *A* astrocytoma, *NA* not available, *O* oligodendroglioma, *OA* oligoastrocytoma, *KPS* Karnofsky performance status, *WHO* World Health Organization.
